# Daucosterol Inhibits the Proliferation, Migration, and Invasion of Hepatocellular Carcinoma Cells via Wnt/β-Catenin Signaling

**DOI:** 10.3390/molecules22060862

**Published:** 2017-06-02

**Authors:** Junquan Zeng, Xing Liu, Xiaofei Li, Yongliang Zheng, Bin Liu, Youzhang Xiao

**Affiliations:** 1 Department of Hematology, Jinggangshan University, Ji’an 343009, China; commandozjq@tom.com; 2 Molecular Biology Laboratory, Jinggangshan University, Ji’an 343009, China; bjxj2005@163.com; 3 Department of Nursing, The Central People’s Hospital of Ji’an City, Ji’an 343000, China; l13576603088@163.com; 4 Department of Hematology, The Affiliated Hospital of Jinggangshan University, Ji’an 343000, China; zyl19891219@163.com (Y.Z.); xiaoyz@jgsu.edu.cn (B.L.); 5 Department of Pathology, Jinggangshan University, Ji’an 343009, China

**Keywords:** daucosterol, Wnt/β-catenin signaling, hepatocellular carcinoma, proliferation, migration, invasion

## Abstract

Hepatocellular carcinoma (HCC) is one of the leading causes of cancer-related death worldwide. The purpose of this study was to determine the effects of daucosterol on HCC by investigating Wnt/β-catenin signaling. In this study, HepG2 and SMMC-7721 cells were treated with varying concentrations of daucosterol, and the corresponding inhibitory effects on HCC cells were examined via CCK-8 assays. Cell migration and invasion abilities were detected via transwell assays. β-Catenin and phospho (p)-β-catenin levels were analyzed via western blotting. Our results showed that daucosterol reduced the proliferation, migration, and invasion capacities of HCC cells in a concentration-dependent manner. In addition, daucosterol reduced the levels of β-catenin and p-β-catenin in HepG2 and SMMC-7721 cells. Furthermore, the Wnt signaling pathway inhibitor SB-216763 was used to treat HepG2 and SMMC-7721 cells with daucosterol. Our results showed that co-treatment with daucosterol and SB-216763 abolished the effects of daucosterol on cell inhibition ratios, cell migration, and cell invasion. These findings indicated that daucosterol inhibited cell migration and invasion in HCC cells via the Wnt/β-catenin signaling pathway. Therefore, our study highlights the use of daucosterol as a promising therapeutic strategy for HCC treatment.

## 1. Introduction

Hepatocellular carcinoma (HCC) ranks as the second major cause of cancer morbidity and mortality worldwide. Each year, about 745,000 new deaths can be attributed to HCC [1,2,3,4], making it a major health problem. HCC mainly results from cirrhosis caused by alcoholic injury, genetic factors, and chronic infections, such as hepatitis B and hepatitis C viral infections [5]. HCC has a low detection rate at the curable stages and has a high rate of recurrence and metastasis, resulting in low survival rates despite recent advancements in therapeutic strategies. Chemotherapy is one of the primary treatment methods for terminal-stage HCC. However, most anticancer drugs exert strong cytotoxic effects and result in the development of drug resistance, thereby limiting its widespread application and curative effects. Accordingly, there is an urgent need to develop more effective treatment methods and identify relevant molecular targets for HCC.

Current studies aim to discover novel drugs that not only significantly inhibit cell proliferation and induce cell apoptosis, but also exert minimal side effects. Natural compounds serve as excellent drug candidates for the treatment of various types of cancers [6,7,8]. Plant-derived sterols (phytosterols) are necessary phytochemicals; to date, about 44 types of phytosterols have been shown to be produced by higher plants, including stigmasterol, β-sitosterol, and campesterol [9,10,11]. Studies have shown that a diet containing 2% mixed phytosterols can inhibit cell proliferation, reduce tumor size, and decrease morbidity and mortality rates in patients with prostate cancer, colorectal cancer, and breast cancer [12,13,14,15]. Daucosterol is one such phytosterol.

Previous studies have shown that daucosterol can inhibit colorectal cancer cell proliferation, migration, and invasion and can induce apoptosis in cancer cells through the caspase signaling pathway [16]. Moreover, daucosterol inhibits cancer cell proliferation and induces autophagy through the generation of reactive oxygen species [17]. An increasing number of studies have shown that daucosterol plays crucial roles in suppressing inflammation and acts as an immunomodulator; daucosterol also increases the proliferative capacity of neural stem cells [18,19,20,21]. However, the anticancer effects of daucosterol in HCC are not entirely understood.

The Wnt family is composed of 19 secreted glycoproteins that are known to play important roles in various biological processes, including cell proliferation, differentiation, migration, and invasion [22]. Wnt proteins can interact with low-density lipoprotein receptor-related protein 5/6 and inhibit the phosphorylation and subsequent degradation of β-catenin. β-Catenin is then translocated to the nucleus, where it influences the function of T-cell factor/lymphoid-enhancing factor (TCF/LEF) in regulating target genes involved in cancer progression [23]. The Wnt/β-catenin signaling pathway plays essential roles in various cancers and is considered a biomarker for hepatic oncogenesis. Further research on the involvement of Wnt signaling in HCC may contribute to the development of more efficacious treatments.

Daucosterol is widely distributed in various plants and can be extracted through a simple process; moreover, daucosterol has been synthetically prepared since 1913. Researchers have also shown that daucosterol is involved in a variety of biological processes, such as development, apoptosis, proliferation, carcinogenesis, and tumor growth [17,21,24]. In this study, we demonstrated the effects of daucosterol on the proliferation, migration, and invasion capacities of HCC cells. Furthermore, we investigated the effects of daucosterol on the expression levels of Wnt/β-catenin signaling proteins in HCC cells. Our findings also demonstrated that daucosterol could serve as a promising therapeutic agent for HCC.

## 2. Results

### 2.1. Daucosterol Inhibited the Proliferation of HepG2 and SMMC-7721 Cells

**Figure 1:**
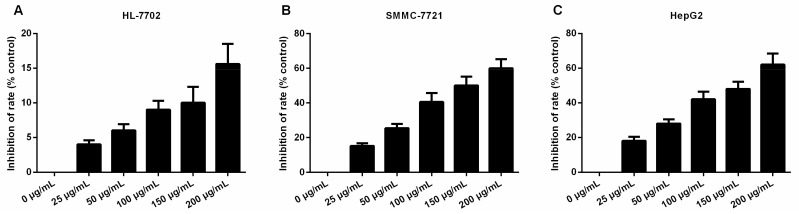
Daucosterol decreased the proliferative capacities of HL-7702, HepG2, and SMMC-7721 cells. CCK-8 assays were performed to determine the inhibition of HL-7702 (**A**); SMMC-7721 (**B**); and HepG2 (**C**) cell proliferation upon treatment with varying concentrations of daucosterol for 48 h.

To investigate the effects of daucosterol on the proliferation of HCC cells, normal liver epithelial (HL-7702) cells, HepG2 cells, and SMMC-7721 cells were treated with varying concentrations of daucosterol (0, 25, 50, 100, 150, and 200 μg/mL) for 48 h. CCK-8 assays were performed to determine the inhibitory effects of the treatments on the HL-7702, HepG2, and SMMC-7721 cells. The results showed that daucosterol significantly inhibited the proliferative ability of HL-7702, SMMC-7721, and HepG2 cells in a concentration-dependent manner, with half-maximal inhibitory (IC_50_) values of 214.99 (Figure 1A), 143.40 (Figure 1B), and 138.73 μg/mL (Figure 1C), respectively. Therefore, these findings suggested that daucosterol significantly suppressed the proliferation ability of HCC cells in a concentration-dependent manner.

### 2.2. Daucosterol Decreased Cell Migration and Invasion Abilities of HepG2 and SMMC-7721 Cells

**Figure 2:**
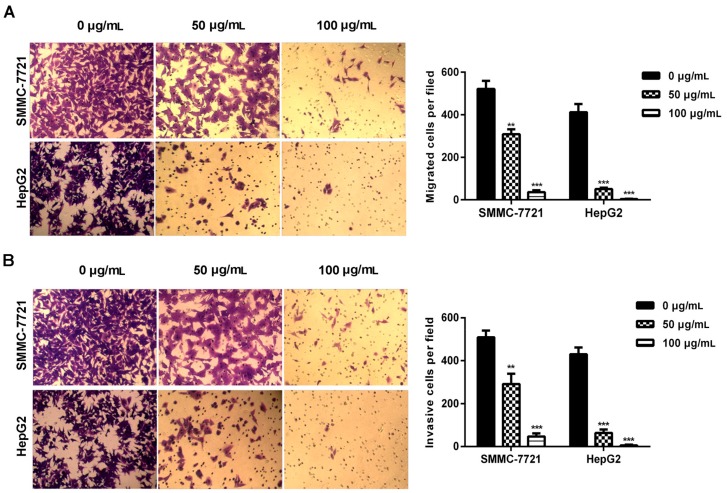
Daucosterol reduced the migration and invasion of HepG2 and SMMC-7721 cells. (**A**) Transwell assays were performed to evaluate the migration of SMMC-7721 and HepG2 cells treated with 0, 50, and 100 μg/mL daucosterol for 48 h (*** *p* < 0.001); (**B**) Cell invasion was detected via transwell assays in SMMC-7721 and HepG2 cells treated with 0, 50, and 100 μg/mL daucosterol for 48 h (** *p* < 0.01, *** *p* < 0.001).

Malignant cancer cells acquired cell migration and invasion capabilities. In the present study, transwell assays were performed to determine the effects of daucosterol on the migration and invasion of HCC cells. Results revealed that daucosterol treatment reduced the migration abilities of HepG2 and SMMC-7721 cells in a concentration-dependent manner (Figure 2A). The invasion of HepG2 and SMMC-7721 cells was also inhibited by daucosterol treatment in a concentration-dependent manner (Figure 2B). These results indicated that daucosterol acted as an inhibitor of HCC cell migration and invasion.

### 2.3. Daucosterol Suppressed the Expression of Wnt/β-Catenin Signaling Proteins in HepG2 and SMMC-7721 Cells

**Figure 3:**
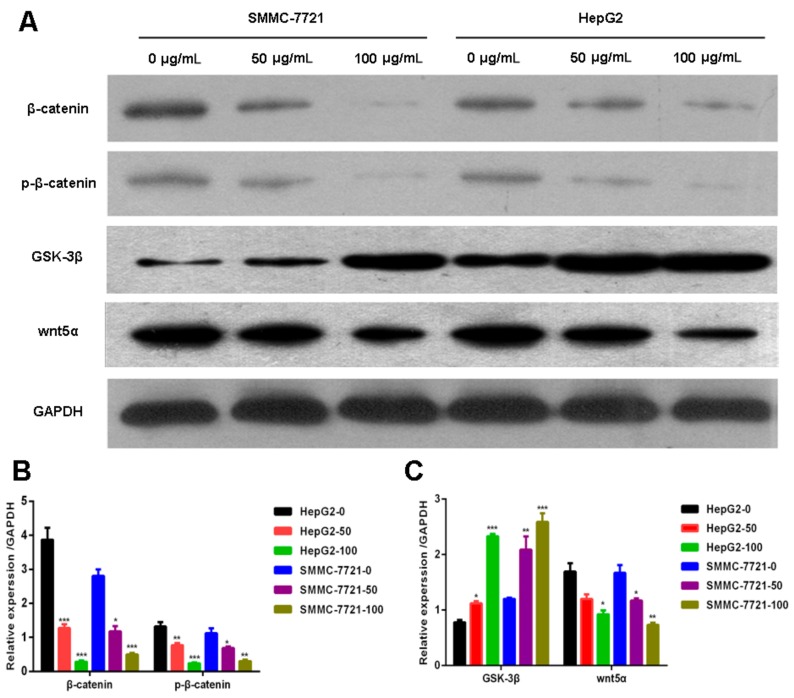
Daucosterol inhibited the expression of Wnt/β-catenin signaling proteins in HepG2 and SMMC-7721 cells. HepG2 and SMMC-7721 cells were treated with 0, 50, and 100 μg/mL daucosterol for 48 h. (**A**) Western blotting was performed to detect the levels of β-catenin, p-β-catenin, GSK-3β, and Wnt5α in treated HepG2 and SMMC-7721 cells. GAPDH was used as a protein loading control; (**B**) Daucosterol reduced β-catenin and p-β-catenin levels in HepG2 and SMMC-7721 cells (* *p* < 0.05, ** *p* < 0.01, *** *p* < 0.001); (**C**) Daucosterol inhibited GSK-3β and Wnt5α expression in HepG2 and SMMC-7721 cells (* *p* < 0.05, ** *p* < 0.01, *** *p* < 0.001).

Wnt signaling, which is induced by β-catenin, is recognized as an important regulator of chemoresistance in numerous tumors, and inhibition of Wnt/β-catenin signaling is known to increase the sensitivity of cancer cells to drugs [25,26,27]. Therefore, we further explored the effects of daucosterol on the expression of Wnt/β-catenin signaling proteins in HCC cells. Western blotting was performed to determine the levels of β-catenin, phospho (p)-β-catenin, glycogen synthase kinase (GSK)-3β, and Wnt5α in treated HepG2 and SMMC-7721 cells. As shown in Figure 3A, HepG2 and SMMC-7721 cells showed decreased β-catenin and phospho-β-catenin levels following daucosterol treatment. Daucosterol significantly reduced β-catenin and phospho-β-catenin levels in both SMMC-7721 and HepG2 cells in a concentration-dependent manner (Figure 3B). Daucosterol significantly reduced Wnt5α expression levels in both SMMC-7721 and HepG2 cells, but increased GSK-3β expression levels (Figure 3C) in a concentration-dependent manner.

### 2.4. Daucosterol Inhibited the Proliferation, Migration, and Invasion of HCC Cells through Wnt/β-Catenin Signaling

**Figure 4:**
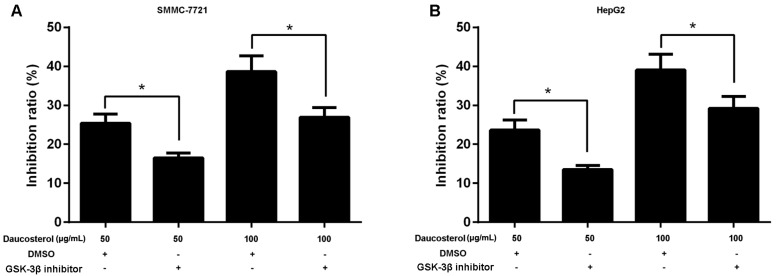
Daucosterol inhibited the proliferation of HCC cells through the Wnt/β-catenin signaling pathway. (**A**) HepG2 and (**B**) SMMC-7721 cells were treated with daucosterol (50 or 100 μg/mL) for 48 h, a Wnt signaling pathway inhibitor (SB-216763, 9 nM) for 48 h, or an equivalent amount of dimethyl sulfoxide (DMSO, control). The cell inhibition ratios in treated SMMC-7721 and HepG2 cells were measured via CCK-8 assays (* *p* < 0.05).

To further evaluate the role of the Wnt-signaling pathway in HCC cell migration and invasion, the Wnt signaling pathway inhibitor SB-216763 was used to investigate the effects of daucosterol on HCC development mediated by Wnt/β-catenin signaling. HepG2 and SMMC-7721 cells were treated with daucosterol (50 or 100 μg/mL) for 48 h, a Wnt signaling pathway inhibitor (SB-216763, 9 nM) for 48 h, or an equivalent amount of dimethyl sulfoxide (DMSO, control). CCK-8 assays were then performed to analyze cell proliferation inhibition ratios in HepG2 and SMMC-7721 cells. The results revealed that daucosterol increased the cell inhibition ratios in SMMC-7721 (Figure 4A) and HepG2 (Figure 4B) cells, whereas co-treatment with daucosterol and SB-216763 abolished the effects of daucosterol on cell inhibition.

**Figure 5:**
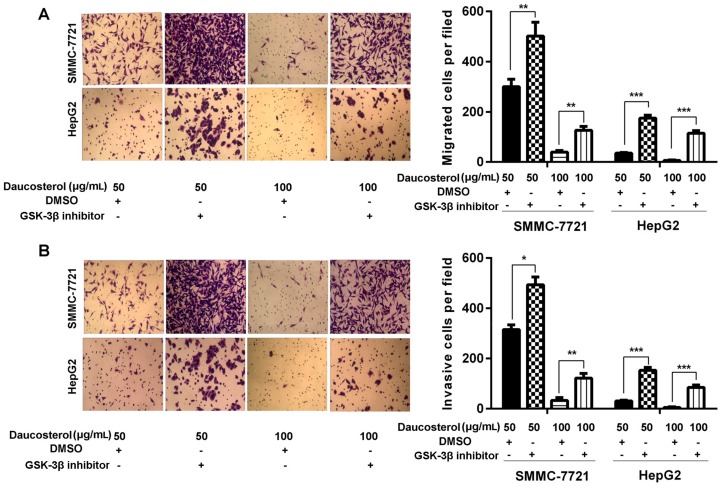
Daucosterol inhibited cell migration and invasion in HCC cells through the Wnt/β-catenin signaling pathway. HepG2 and SMMC-7721 cells were treated with daucosterol (50 or 100 μg/mL) for 48 h, a Wnt signaling pathway inhibitor (SB-216763, 9 nM) for 48 h, or an equivalent amount of dimethyl sulfoxide (DMSO, control). (**A**) Transwell assays were performed to determine the cell migration abilities of the treated SMMC-7721 and HepG2 cells. Magnification: 200×. Scale bars = 10 μm (** *p* < 0.01, *** *p* < 0.001); (**B**) Transwell assays were performed to determine the invasion abilities of the treated SMMC-7721 and HepG2 cells. Magnification: 200×. Scale bars = 10 μm, (* *p* < 0.05, ** *p* < 0.01, *** *p* < 0.001).

Furthermore, transwell assays were performed to determine the migration and invasion abilities of treated HepG2 and SMMC-7721 cells. The results revealed that daucosterol treatment suppressed the migration of SMMC-7721 and HepG2 cells, whereas co-treatment with both daucosterol and SB-216763 dramatically reversed the inhibitory effects of daucosterol on cell migration (Figure 5A). Furthermore, daucosterol also significantly reduced the invasion of SMMC-7721 and HepG2 cells, whereas co-treatment with both daucosterol and SB-216763 dramatically abrogated the inhibitory effects of daucosterol on cell invasion behavior (Figure 5B).

## 3. Discussion

Natural products have recently attracted the interest of researchers in the field of drug discovery because of the diverse compounds that they produce. Successful application of natural products and their derivatives in anticancer treatment have already been demonstrated [28,29]. Therefore, natural products, particularly plants, represent a novel and effective source of anticancer agents with fewer side effects. Natural compounds have become a major source of conventional medicinal products for use in the treatment of various types of cancers [30,31,32]. Daucosterol—also known as eleutheroside A,β-sitosterol, or β-d-glucoside—is a plant-derived compound that is similar to animal cholesterol [33]. Multiple studies have shown that daucosterol exerts significant biological effects—such as immunomodulatory, anti-inflammatory, and chemopreventive effects—suggesting its potential use as an effective natural component in chemotherapeutic drugs applied in cancer treatment. One study also showed that daucosterol inhibited proliferation and induced apoptosis in breast cancer cells [34]. However, the mechanisms through which daucosterol induces HCC cell death are not fully understood.

In our study, we demonstrated that daucosterol treatment significantly inhibited the proliferation of HepG2 and SMMC-7721 cells in a time- and concentration-dependent manner. In addition, our findings revealed that daucosterol significantly inhibited the migration and invasion of HepG2 and SMMC-7721 cells in a time- and concentration-dependent manner. Consistent with our present findings, a recent study showed that daucosterol inhibits the proliferation, migration, and invasion of colon cancer cells and induces apoptosis by targeting caspase signaling [16]. Further experiments demonstrated that daucosterol treatment results in inactivation of the phosphoinositol 3-kinase/Akt pathway and upregulation of the phosphatase and tensin homolog gene to induce apoptosis in human breast adenocarcinoma cells [35]. Therefore, daucosterol could serve as a potential therapeutic drug for the treatment of HCC. However, further studies are needed to validate the roles of daucosterol in different HCC cell lines, such as Hep-3B, QGY-7703, Huh7, and QGY-7703 cells.

The Wnt/β-catenin signaling network is a crucial regulator of various physiological processes, including cell proliferation, apoptosis, cycle progression, differentiation, transcription, and translation [36,37,38,39]. Accumulating evidence suggests that the activated Wnt/β-catenin pathway provides basic survival signals to many cancer cells. Wnt signaling promotes breast cancer progression by inhibiting ITCH-mediated degradation of WW domain binding protein 2 [40]. The Wnt/β-catenin pathway is also known to induce the epithelial-to-mesenchymal transition in breast cancer cells [41] and accelerate colorectal cancer tumorigenesis and progression by regulating SATB homeobox 1 [42]. Therefore, the Wnt/β-catenin pathway represents an important research topic for developing treatment strategies for patients with various types of cancers.

GSK-3 is also a major component of the Wnt-signaling pathway [43]. Some GSK-3 is closely related to the axin-scaffolding protein, which binds adenomatous polyposis coli and β-catenin during the period of dormancy [44]. GSK-3-mediated phosphorylation of β-catenin targets leads to ubiquitination and proteosome-mediated degradation [45]. 3-(2,4-Dichlorophenyl)-4-(1-methyl-1*H*-indol-3-yl)-1*H*-pyrrole-2,5-dione (SB216763) is a potent, selective GSK-3 inhibitor that competes with ATP. Studies have shown that SB216763 has significant therapeutic effects in pulmonary inflammation and fibrosis [46]. In our study, we used the Wnt signaling pathway inhibitor SB-216763 to inhibit GSK3 expression and subsequently suppress β-catenin expression.

In the present study, we measured the levels of β-catenin and p-β-catenin in HepG2 and SMMC-7721 cells treated with daucosterol and showed that daucosterol significantly decreased β-catenin and p-β-catenin levels in HepG2 and SMMC-7721 cells in a concentration-dependent manner. Daucosterol significantly increased GSK-3β expression and significantly decreased Wnt5α expression in a concentration-dependent manner. Next, we used the Wnt signaling pathway inhibitor SB-216763 to study the effects of daucosterol on HCC progression mediated by Wnt/β-catenin signaling. The results suggested that daucosterol inhibited the migration and invasion of HCC cells through Wnt/β-catenin signaling. Similarly, current studies have also suggested that the Wnt/β-catenin signaling pathway promotes the hypoxia-induced epithelial-to-mesenchymal transition in HCC cells [47]. The Wnt/β-catenin signaling pathway has been reported to influence angiogenic growth factors involved in HCC [48]. Moreover, *miR-429* has been shown to promote HCC metastasis through the Wnt pathway [49]. These findings imply that the Wnt/β-catenin signaling pathway significantly influences the migration and invasion abilities of HCC.

In summary, our results showed that daucosterol inhibited the proliferation, migration, and invasion capacities of HCC cells in a time- and concentration-dependent manner. Furthermore, daucosterol was found to suppress the expression of Wnt/β-catenin signaling proteins in HCC cells. Daucosterol treatment reduced the migration and invasion abilities of HCC cells through the Wnt/β-catenin signal pathway. Our findings indicated that daucosterol was a promising candidate for use in the therapeutic treatment of HCC. Therefore, daucosterol could serve as an efficient and inexpensive growth factor and could have applications in clinical medicine and research applications. However, further studies using nude mice are required to validate these in vitro observations. Moreover, the exact mechanisms underlying the effects of daucosterol through Wnt/β-catenin signaling remain to be elucidated.

## 4. Materials and Methods

### 4.1. Cell Culture and Treatment

Human HCC cell lines HepG2 and SMMC-7721 were purchased from the Cell Bank of Chinese Academy of Sciences (Shanghai, China). HepG2 and SMMC-7721 cells were grown in Dulbecco’s Modified Eagle’s Medium (DMEM; Invitrogen, Carlsbad, CA, USA) containing 10% fetal bovine serum (FBS; Gibco, Grand Island, NY, USA), 100 U/mL penicillin, and 1 μg/mL streptomycin (Invitrogen) in an incubator with 5% CO_2_ at 37 °C. The culture medium was changed daily.

HepG2 and SMMC-7721 cells were seeded in 6-well plates (1 × 10^5^ cells/well) and treated with varying concentrations of daucosterol (0, 25, 50, 100, 150, and 200 μg/mL) for 48 h. HepG2 and SMMC-7721 cells were treated with daucosterol (50 or 100 μg/mL) for 48 h, a Wnt signaling pathway inhibitor (SB-216763, 9 nM) for 48 h, or DMSO (control).

### 4.2. Proliferation Assay

Cell proliferation was measured via cholecystokinin octapeptide (CCK-8) assays. Treated HepG2 and SMMC-7721 cells were seeded in 96-well plates containing 100 μL DMEM (10% FBS) at a density of 2 × 10^3^ cells/well and cultured in an incubator with 5% CO_2_ at 37 °C for 48 h. Ten microliters of WST (Dojindo Laboratories, Kumamoto, Japan) was added to each well at a specific time point. After 4 h, absorbance values at 450 nm were recorded using an Elx800 Reader (Bio-Tek Instruments Inc., Winooski, VT, USA).

### 4.3. Transwell Assays

In vitro cell migration and invasion of HCC cells were detected in 24-well transwell cell culture chambers (Costar) according to the manufacturer’s instructions. For migration assays, treated HepG2 and SMMC-7721 cells (3 × 10^3^ cells/well) were seeded in the upper chamber of uncoated transwell inserts (Millipore, Billerica, MA, USA) without serum. For the invasion assay, 50 μL of 2.0 mg/mL Matrigel (BD Biosciences) was plated on the upper chamber of uncoated transwell inserts for 30 min at 37 °C. Similarly, 3 × 10^3^ cells were seeded in the upper chambers of the transwell inserts without serum. Additionally, 600 μL culture medium with 10% FBS was added to the lower chambers and served as a chemoattractant to induce cell migration. After 24 h, cells that did not migrate or invade the chamber were removed using a cotton swab. Migratory and invasive cells were fixed with 4% paraformaldehyde, stained with crystal violet, and photographed under a phase-contrast microscope (Olympus, Tokyo, Japan). Five random views were selected for cell counting. In addition, we calculated the migration and invasion of the cells on the basis of the actually migratory and invasive cells and the cell survival rate, as follows: migratory and invasive cells = the actually migratory and invasive cells/cell survival rate.

### 4.4. Western Blot Analysis

Treated HepG2 and SMMC-7721 cells were lysed using lysis buffer containing a protease inhibitor cocktail (P8340; Sigma-Aldrich, St. Louis, MO, USA). Total protein concentrations were analyzed using a BCA Protein Assay kit (Thermo Fisher Scientific, Rockford, IL, USA). Heat-denatured proteins (30 μg) were separated by sodium dodecyl sulfate polyacrylamide gel electrophoresis based on molecular weight using 8% gels and subsequently transferred to polyvinylidene difluoride membranes (Millipore). The transferred proteins were then blocked using 5% skim milk (BD Biosciences) for 2 h at room temperature and then incubated with primary antibodies (anti-β-catenin and anti-(p)-β-catenin) at the appropriate dilution at 4 °C overnight. The next day, membranes were incubated with the secondary antibody. Signals were detected using an enhanced chemiluminescence (ECL) substrate kit (Amersham Biosciences, Inc., Piscataway, NJ, USA) and detection system (Amersham Biosciences). Mouse anti-β-catenin (1:1000; BD Transduction Laboratories, San Jose, CA, USA) and mouse anti-(p)-β-catenin antibodies (1:1000; Cambridge, MA, USA) were used as the primary antibodies. Mouse anti-GAPDH monoclonal antibodies (1:5000; Cell Signaling Technology, Beverly, MA, USA) were used as an internal control.

### 4.5. Statistical Analysis

Data were analyzed by one-way analysis of variance using SPSS (IBM SPSS Statistics 20; Chicago, IL, USA). All experiments were performed at least three times. All results were counted and presented as means ± standard deviations. Differences with *p* values of less than 0.05 were considered statistically significant.
